# Methylphenidate use and misuse among medical residents in Israel: a cross-sectional study

**DOI:** 10.1186/s12960-023-00792-x

**Published:** 2023-01-31

**Authors:** Eden Zahavi, Liat Lev-Shalem, Ilan Yehoshua, Limor Adler

**Affiliations:** 1 Health Division, Maccabi Healthcare Services, 27 Ha’mered St., Tel Aviv, Israel; 2 Faculty of Health Sciences, Siaal Research Center for Family Medicine and Primary Care, Ben-Gurion University of the Negev, Beer-Sheva, Israel; 3 Department of Family Medicine, Sackler Faculty of Medicine, Tel Aviv, Israel

**Keywords:** Methylphenidate, Misuse, Cognitive enhancers, Medical residents, ADHD, Non-medical use of prescription stimulants

## Abstract

**Background:**

Methylphenidate (MPH) and other stimulants may be misused, mainly as cognitive enhancers and recreational drugs. Data regarding misuse among medical residents are scarce. This study aimed to evaluate the prevalence of and main reasons for methylphenidate (MPH) use and misuse among Israeli medical residents.

**Methods:**

In this cross-sectional study, we sent an online questionnaire to medical residents who had completed their first residency exam and specialists with up to 2 years of experience. We asked about the use of MPH before and during residency and attitudes toward the use of MPH as a cognitive enhancer. We also added the Adult ADHD Self-Report Scale (ASRS) questionnaire, a validated tool used to screen for the presence of attention deficit hyperactivity disorder (ADHD). Users and misusers were classified based on self-report of use and formal ADHD diagnosis. Logistic regression analysis was used to evaluate factors associated with MPH misuse.

**Results:**

From March 2021 to August 2021, 370 physicians responded to our questionnaire (response rate 26.4%). Twenty-eight met the exclusion criteria and were not included. The respondents’ average age was 36.5 years. Women comprised 63.5% of the respondents. Of the participants, 16.4% were classified as users and 35.1% as misusers. The prevalence of misusers was 45.6% among surgery and OB/GYN physicians, 39.4% among pediatricians and internists, and 24% among family physicians (*P* < 0.001). Misusers had a more liberal approach than others to MPH use as a cognitive enhancer. Factors associated with misuse of MPH included not being a native-born Israeli (OR-1.99, 95% CI 1.08, 3.67) and type of residency (OR-2.33, 95% CI 1.22, 4.44 and OR-4.08, 95% CI 2.06, 8.07 for pediatrics and internal medicine and surgery, respectively).

**Conclusion:**

Very high levels of MPH misuse during residency may be related to stress, long working hours, night shifts, and the academic burden of the residency period. We believe that our findings should be considered by healthcare policymakers as they make decisions regarding the conditions of medical residencies. The use of MPH as a cognitive enhancer should be further studied and discussed.

**Supplementary Information:**

The online version contains supplementary material available at 10.1186/s12960-023-00792-x.

## Background

Methylphenidate (MPH) is a CNS stimulant that acts on the brainstem arousal system and cortex. Short- and long-acting formulations of MPH and other stimulants have been approved by the United States (US) Food and Drug Administration (FDA) for use in the treatment of attention deficit hyperactivity disorder (ADHD) and narcolepsy [1]. The total usage rate of MPH in all industrialized countries has increased significantly in the last several years. In the US, the rate of stimulant dispensing increased significantly between 2014 and 2019 and stood at 6.1 prescriptions per 100 persons in 2019 [2].

### Misuse of MPH

In recent years, MPH and other stimulants have begun to be misused, mainly as cognitive enhancers and recreational drugs [3]. Misuse or nonmedical use occurs when a licit medication is used for a purpose for which it was not prescribed to elicit a nontherapeutic or nonmedical effect, e.g., performance enhancement. The rate of stimulant misuse in adults in the US between 2015 and 2016 was estimated at approximately 2% [4] and has increased substantially each year since then [5]. Among students, MPH is one of the most popular cognitive enhancers [6]. The rate of MPH misuse in college students is estimated at 17% [7]. Several studies have demonstrated that the main motivation for misusing prescription stimulants is cognitive and academic enhancement [7–9].

A great deal of research has been conducted on the cognitive effects of stimulants and their potential as neurocognitive enhancers [10–15]. In a recent review, the effects of MPH were revealed to be improvement in working memory and processing speed, well-being (as a possible cognitive performance booster), recall, and sustained attention, as well as positive impacts on fatigue and declarative memory [16].

MPH is considered to have a relatively good safety profile at formal dosages, but its adverse effects include insomnia, anorexia, dry mouth, abdominal pain, palpitations, and headache [16, 18]. Some of its more troubling side effects include mood lability [17], and, in extreme cases, even psychosis [18]. The rapid expansion of methylphenidate analogs into the drug market in recent years has led to several occurrences of intoxication, some of which resulted in fatalities [19]. Misuse of MPH is considered a significant public health problem, especially among students [20].

### Misuse of MPH in the medical profession

Medical students and residents are an interesting subpopulation in terms of the misuse of MPH and other stimulants. On the one hand, their academic loads are significant and include large numbers of exams and tasks that must be completed under time pressure. On the other, their access to knowledge regarding the side effects and unwanted results of stimulant drug use should enable them to make informed choices on the matter.

Several studies have examined patterns of MPH use and misuse in medical students. The range of MPH use was 8% to 17%, with most studies pointing to its use in the absence of a formal diagnosis of ADHD to enhance academic performance [21–26]. Cohen YG showed that 17% of Israeli medical students, in their cohort, had used MPH at least once, among them 48.7% without prescription. In this study, the use of MPH increased consistently from 6% in the first year to 18% in the sixth year [26]. Finger and colleagues demonstrated that usage initiation was seen mainly during the academic year for academic score improvement [27]. In the same study, it was also found that competition and stress were associated with the misuse of MPH among medical students. De Bruyn et al. demonstrated an association between competition, stress, and misuse of prescription stimulants among medical students [28].

While much research has been conducted on medical students, data regarding medical residents are scarce. Residents are often under cognitive and emotional stress due to long hours at the hospital, including night shifts, in addition to academic tasks and exams. Moreover, most residents are at the stage of life when they are establishing families and raising children. This study aimed to evaluate stimulant drug use and misuse rates among Israeli medical residents and shed light on the main reasons for the use and misuse of MPH in this population.

## Methods

### Setting and study design

We conducted a descriptive cross-sectional survey among residents who ranged from those who had taken their first residency exams to specialists who had completed their residencies up to 2 years previously. We used a convenient sample of residents from different specialties. We sent an online questionnaire via e-mail, text message, and social media (groups of residents on Facebook). We also used the snowball method for recruitment and asked residents to send the questionnaire to groups of their fellow residents (through email and WhatsApp groups). In this way, we reached residents from different hospitals and departments throughout the country. The questionnaire was administered in Hebrew. An English translation is available as Additional file 1.

We asked the respondents about their use of MPH before and during their residencies and their attitudes toward the use of MPH as a cognitive enhancer. We formulated the statements regarding attitudes toward MPH misuse because we could not locate a previous questionnaire on which to rely. We also asked the respondents to fill out the Adult ADHD Self-Report Scale (ASRS) questionnaire. The ASRS is a questionnaire developed by the World Health Organization (WHO) to facilitate screening for ADHD [29, 30]. It originally consisted of 18 questions, but later six were chosen to optimize the clinical classification. The Hebrew questionnaire was validated in 2010 [31]. We collected data on sociodemographic parameters, including age, gender, country of birth, native language, number of children, and type of residency. Several reminders were sent to the physicians.

Participation in the study was voluntary and anonymous. We stated in the questionnaire that each resident could answer the questionnaire only once, but we did not have any technical way to ensure that this would be the case. Consent to participate was granted by submission of a completed questionnaire. The study was approved by the institutional review board of Maccabi Healthcare Services (0126-20-MHS).

### Variables

Respondents were classified as users, misusers, or non-users based on their answers. Physicians who used MPH and were formally diagnosed with ADHD were classified as users, those who used MPH without a formal diagnosis (regardless of their ASRS score) were classified as misusers, and all the rest were classified as non-users.

The ASRS was defined as positive or negative, reflecting the presence or absence of ADHD, respectively. All items that represented attitudes towards MPH usage were ranked using a 5-point Likert scale (1 = strongly disagree, 5 = strongly agree), with higher scores representing a relatively conservative approach and lower scores representing a relatively liberal one.

### Sample size

We calculated the sample size according to the main aim of this study, which was to evaluate the prevalence of MPH misuse among residents in Israel. Our initial hypothesis was that the rate of misuse would be 20%, with a 5% acceptable difference and a 95% confidence interval. Based on this assumption, we calculated a sample size of 309 participants using WinPepi. To compare the prevalence of MPH misuse among different groups of residents, we assumed a proportion of 20% in one group and 40% in the second group. For this, we required a sample of at least 91 residents in each group, with a 5% significance level and 80% power.

### Statistical analysis

The sociodemographic characteristics of the participants and the patterns of their MPH usage were presented in absolute numbers and percentages. Differences between groups of users (misusers, users, and non-users) were analyzed by means of Chi-square tests for categorical variables and ANOVA for continuous variables.

We used the ANOVA test to evaluate differences in attitudes between users, misusers, and non-users of MPH. The Bonferroni correction was used to detect the source of differences between the groups. We performed a multivariate analysis to evaluate the factors associated with being a misuser using logistic regression with a forward approach. In the multivariate regression, we also assessed whether interactions existed. Users were not included in this analysis. All analyses were performed using IBM SPSS v.27®.

## Results

### Study population

From March 2021 to August 2021, we sent the questionnaire to 1 400 physicians from different specialties. Three hundred seventy physicians responded (26.4% response rate). Among the respondents were family physicians, pediatricians, internists, and surgeons (general, orthopedic, ENT, plastic, cardiothoracic, and OB/GYN). Twenty-eight did not take part because they met the exclusion criteria, not having taken their first residency examination. The average age of respondents was 36.5 years. Two hundred seventeen (63.5%) were women and 279 (81.6%) were native-born Israelis (Table 1). Of all the respondents who met the inclusion criteria, 57 (16.7%) reported having been formally diagnosed with ADHD, and 93 (27.2%) screened positive based on the ASRS.

**Table 1:** Characteristics of physicians who participated in the study

	Mean (SD)
Age	36.5 (3.35) Range: 28–51

	*n* (%)
Gender	
Female	217 (63.5)
Country of birth	
Israel	279 (81.6)
Native language	
Hebrew	263 (76.9)
Number of children	
0	62 (18.1)
1–3	280 (81.9)
Residency	
Surgery	90 (26.3)
Pediatrics/internal medicine	99 (28.9)
Family medicine	96 (28.1)
Other	57 (16.7)
Past diagnosis with ADHD	
Yes	57 (16.7)
ASRS	
Positive	93 (27.2)
Use of MPH (ever)	
Yes	176 (51.5)
Use of MPH (during residency)	
Yes	172 (50.3)
First use of MPH	
School	11 (6.3)
Pre-clinical years of medical school	40 (22.7)
Clinical years of medical school	41 (23.3)
Residency	84 (47.7)

### Main results

#### Misuse of methylphenidate in Israel

Of the 342 respondents, 176 (51.5%) reported using MPH. One hundred twenty (120/342; 35.1% of the study population) had no official medical diagnosis of ADHD and were thus defined as misusers. Based on the ASRS self-questionnaire, 249 (72.8%) screened negative for ADHD, yet 113 of them (45.4%) reported using MPH. One hundred fifty-four (87.5%) of all MPH users reported using MPH for final residency exam preparation, and 33 (18.7%) used it during night shifts. Frequency of usage varied between occasional (on an “as needed” basis) to daily use (Table 2). Most MPH users’ first use took place in the preclinical and clinical years, when they were medical students (42.9%), while most misusers’ first use took place during residency (58.3%) (*P* < 0.001). A native language other than Hebrew was associated with misuse of MPH, while more native Hebrew speakers were classified as users (*P*-0.026). We found no significant differences in the age, gender, or country of birth of users, misusers, and non-users. Fifty-five percent of misusers, 85.7% of users, and 45.2% of non-users reported that they were familiar or very familiar with the safety profile and adverse effects of MPH (*P* < 0.001).

**Table 2:** Univariate comparison of respondents stratified by use of MPH

	Misusers *N* = 120	Users *N* = 56	Non-users *N* = 166	*P* value
Age				
Mean (SD)	36.2 (3.48)	37.3 (2.99)	36.4 (3.35)	0.134

	*n* (%)	*n* (%)	*n* (%)	
Gender				
Female	71 (32.7)	34 (15.7)	112 (51.6)	0.317
Male	49 (39.2)	22 (17.6)	54 (43.2)	
Country of birth				
Israel	90 (32.3)	49 (17.6)	140 (50.2)	0.064
Other	30 (47.6)	7 (11.1)	26 (41.3)	
Native language				
Hebrew	85 (32.3)	50 (19)	128 (48.7)	0.026
Other	35 (44.3)	6 (7.6)	38 (48.1)	
Residency				
Surgery	41 (45.6)	20 (22.2)	29 (32.2)	
Pediatrics/internal medicine	39 (39.4)	13 (13.1)	47 (47.5)	< 0.001
Family medicine	23 (24)	10 (10.4)	63 (65.6)	
Other	17 (29.8)	13 (22.8)	27 (47.4)	
ASRS—positive	29 (24.2)	34 (60.7)	30 (18.1)	< 0.001
First use of MPH				
Pre-clinical years of medical school	17 (14.2)	34 (60.7)		
Clinical years of medical school	33 (27.5)	8 (14.3)		< 0.001
Residency	70 (58.3)	14 (25)		
Frequency of MPH use				
As needed	46 (38.3)	9 (16.1)		
Daily (< 1 month)	32 (26.7)	7 (12.5)		
Daily (≥ 1 month)	32 (26.7)	35 (62.5)		< 0.001
No response	10 (8.3)	5 (8.9)		
Use for exams				
Yes	106 (88.3)	48 (85.7)		0.807
Average attitude score^a^				
Mean (SD)	2.23 (0.74)	2.72 (0.86)	3.40 (0.80)	< 0.001

^a^Using Bonferroni correction, the difference was < 0.001 for all comparisons

#### Misuse of methylphenidate in different specialties

Forty-one (45.6%) surgery and OB/GYN physicians, 39 (39.4%) pediatricians and internists, and 23 (24%) family physicians reported using MPH without an official diagnosis of ADHD (*P* < 0.001) (Table 2).

#### Attitudes

The Cronbach alpha was calculated for the attitude items and showed good internal validity (Cronbach alpha = 0.79). Average scores were 2.23 ± 0.74, 2.72 ± 0.86, and 3.40 ± 0.8 for misusers, users, and non-users, respectively. Non-users (compared to users and, to a greater extent, compared to misusers) objected more to the use of MPH as a cognitive enhancer and agreed more with the following statements: the use of MPH without a formal diagnosis promotes unfairness, MPH should be given only when there is a fundamental impact on functioning, and first prescription and changes in MPH prescriptions should be made only by a physician certified in the field of ADHD.

#### Factors associated with misuse of MPH

In a multivariate analysis, misuse was associated with not being a native-born Israeli (OR-1.99, 95% CI 1.06, 3.73) and with residency type (OR-5.61, 95% CI 2.17, 14.5 and OR-4.4, 95% CI 1.82, 10.63 for pediatrics and internal medicine and surgery, respectively) (Table 3). When assessing the influence of interactions between variables, we discovered that gender and residency interact; female residents in pediatrics and internal medicine had lower odds of being misusers (OR-0.28, 95% CI 0.11, 0.74). All other variables (age, gender, native language, number of children, ASRS, and the interaction between age and country of birth were not found to be significant and were not included in the model (using the forward approach).

**Table 3:** Multivariate analysis of MPH misusers vs. non-users (forward method)

	Odds ratio (95% confidence interval)	*P* value
Country of birth		
Israel	Reference	
Other	1.99 (1.06, 3.73)	0.027
Residency		
Family	Reference	
Pediatric/internal medicine	5.61 (2.17, 14.5)	< 0.001
Surgery	4.4 (1.82, 10.63)	0.001
Other	0.75 (0.19, 2.95)	0.755
Residency* gender		
Pediatric/internal medicine* female	0.28 (0.11, 0.74)	0.011
Surgery* female	0.88 (0.33, 2.31)	0.79
Other* female	3.03 (0.69, 13.32)	0.141

Variables not included in the final model: age group, gender, native language, number of children, ASRS, age group*country of birth

## Discussion

### Main results

In this cross-sectional study, we suggest that the prevalence of MPH use and misuse among Israeli medical residents was 51.5% and 35.1%, respectively. Half of all users in our study began to use MPH during their residencies, and 83.3% used MPH without an official ADHD diagnosis. The main reason for MPH use was preparing for residency examinations (87.5%). Hospital residents and those in the surgical professions (45.6%) were more likely to misuse MPH than family medicine residents (24%). Female pediatricians and internists were less likely to misuse MPH. Those born outside of Israel were more likely than native-born Israelis to misuse MPH.

Misusers and users were more liberal than non-users in their approach toward using and prescribing MPH. In addition, misusers did not perceive the act of misusing as affecting the equality or fairness of exams or as a crossing of boundaries.

### Interpretation

In this study, we suggest that misuse of MPH among Israeli medical residents (35.1%) is extremely high compared to the known misuse rates among the general adult population (2%) [4], college students (17%), and medical students (8–17%) [7].

Twenty-four percent (24%) of respondents reported they began using MPH before residency. This rate is somewhat higher than the rates found in previous studies on medical students. Cohen et al. found a 17% usage rate among Israeli medical students [26].

Interestingly, almost half of all users (84/176, 47.7%) began using MPH during their residencies. The majority of these did not have a formal ADHD diagnosis (70/84, 83.3%) and could be labeled as misusers. The high rate of misuse during residency was associated in our study with preparing for exams (88% of misusers reported using MPH during exam preparation) and working night shifts (22% of misusers reported using MPH during night shifts). These findings are in line with those regarding medical students, among whom the main reasons for misuse of MPH were the desire to enhance academic performance and increase wakefulness [32].

Psychological factors associated with the misuse of MPH include procrastination, difficulty with time management [33], anxiety, and stress [8, 34, 35], all of which characterize the residency period [36–40], a stressful, overwhelming time during which residents work long hours and the lives of others depend on them [41].

The residency period is considered particularly demanding in terms of both tasks and working hours. A recent survey conducted by the Israeli residents’ organization, Mirsham, revealed that most residents work over 280 h a month and many of them are on in-house duty for 26-h shifts six or more times a month [42].

The difference between specialties is the main finding of our study. An especially high rate of MPH misuse was found in residents working in the hospital, specifically in surgical fields. This finding can be explained by the significant variations in the number of hours and night shifts residents from different specialties are required to work. In addition, surgical residents experience more burnout than residents in non-surgical residencies [43].

Another explanation for high rates of misuse, which in many studies reached over 50% [44, 45], could be residents’ tendency to self-prescribe and self-treat. In general, it was found that physicians are vulnerable to substance abuse/addiction due to their access to the substances of abuse. The majority of substance-abusing doctors prescribe drugs to themselves [46]. Self-prescription is an ethical issue that should be addressed by health policymakers, but there are currently no guidelines regarding it or laws against it in Israel, and it is very common [47].

We found that not being a native-born Israeli was associated with MPH misuse. This finding is in line with those of previous studies that revealed immigration as a risk factor for substance abuse [48–50]. It is not surprising to discover that the misusers’ group had the most liberal approach toward prescribing and using stimulant drugs as cognitive enhancers. Two of the factors associated with increased MPH use among students are the perceived safety of the medication and the perception that MPH use is normative on campus (and perhaps during residency) [33, 35].

### Strengths and limitations

One of the main strengths of this study is its nationwide coverage of residents from different medical specialties. To the best of our knowledge, until now, little has been known about the prevalence of MPH misuse among residents.

The main limitations of the study include the low response rate (26.4%), although this rate is not very low compared to other online questionnaire studies, and a possible selection bias. Due to the sampling methods (a convenience and snowball sampling), residents who were more interested than others in the survey subject because they used or misused stimulants may have been more likely to respond, resulting in an overestimation of the apparent involvement of users and misusers in the study. This results in a possible selection bias. The demographic characteristics of the residents who participated in the study are somewhat different from those of the general resident population, and the study included relatively high percentages of women (63.5%) and native-born Israelis (81.6%).

## Conclusion

In this study, we found a very high percentage of MPH misusers (35.1%) among Israeli medical residents, especially hospital and surgical residents. We attribute this high percentage to stress, the burdens of the medical profession, long working hours, night shifts, and academic demands, all of which characterize the residency period in general and in Israel in particular. We also found not being a native-born Israeli to be a risk factor, as it is for misuse of other substances.

We believe that healthcare policymakers should consider our findings when discussing residency conditions. Residents’ employers should be aware of the high rate of MPH misuse and attempt to address this issue, especially in more at-risk groups, including residents in surgical specialties and non-native-born residents. The fact that our study and many others have found that the use of MPH as a cognitive enhancer is widespread among students and residents indicates that the time has come for the medical community to develop intense research on this phenomenon, which deserves to be further explored and discussed. Future studies should explore the prevalence of MPH misuse in a representative sample of residents to eliminate possible selection bias.

## Supplementary Information

**Additional file 1.** Stimulant medication use among Israeli medical residents: questionnaire.

## Data Availability

Data are available from the corresponding author upon reasonable request.

## Abbreviations

ASRS: Adult ADHD Self-Report Scale
ADHD: Attention deficit hyperactivity disorder
CNS: Central nervous system
FDA: Food and Drug Administration
WHO: World Health Organization
MPH: Methylphenidate

## References

[1] Leonard BE, McCartan D, White J, King DJ (2004). Methylphenidate: a review of its neuropharmacological, neuropsychological and adverse clinical effects. Hum Psychopharmacol.

[2] Board AR, Guy G, Jones CM, Hoots B (2020). Trends in stimulant dispensing by age, sex, state of residence, and prescriber specialty—United States, 2014–2019. Drug Alcohol Depend.

[3] Clemow DB (2017). Misuse of methylphenidate. Curr Top Behav Neurosci.

[4] Compton WM, Han B, Blanco C, Johnson K, Jones CM (2018). Prevalence and correlates of prescription stimulant use, misuse, use disorders, and motivations for misuse among adults in the United States. Am J Psychiatry.

[5] Chen L-Y, Crum RM, Strain EC, Alexander GC, Kaufmann C, Mojtabai R (2016). Prescriptions, nonmedical use, and emergency department visits involving prescription stimulants. J Clin Psychiatry.

[6] Sharif S, Guirguis A, Fergus S, Schifano F (2021). The use and impact of cognitive enhancers among university students: a systematic review. Brain Sci.

[7] Benson K, Flory K, Humphreys KL, Lee SS (2015). Misuse of stimulant medication among college students: a comprehensive review and meta-analysis. Clin Child Fam Psychol Rev.

[8] Weyandt LL, Janusis G, Wilson KG, Verdi G, Paquin G, Lopes J (2009). Nonmedical prescription stimulant use among a sample of college students: relationship with psychological variables. J Atten Disord.

[9] Rabiner DL, Anastopoulos AD, Costello EJ, Hoyle RH, McCabe SE, Swartzwelder HS (2009). Motives and perceived consequences of nonmedical ADHD medication use by college students: are students treating themselves for attention problems?. J Atten Disord.

[10] Coghill D, Banaschewski T, Zuddas A, Pelaz A, Gagliano A, Doepfner M (2013). Long-acting methylphenidate formulations in the treatment of attention-deficit/hyperactivity disorder: a systematic review of head-to-head studies. BMC Psychiatry.

[11] Marraccini ME, Weyandt LL, Rossi JS, Gudmundsdottir BG (2016). Neurocognitive enhancement or impairment? A systematic meta-analysis of prescription stimulant effects on processing speed, decision-making, planning, and cognitive perseveration. Exp Clin Psychopharmacol.

[12] Ilieva IP, Hook CJ, Farah MJ (2015). Prescription stimulants’ effects on healthy inhibitory control, working memory, and episodic memory: a meta-analysis. J Cogn Neurosci.

[13] Repantis D, Schlattmann P, Laisney O, Heuser I (2010). Modafinil and methylphenidate for neuroenhancement in healthy individuals: a systematic review. Pharmacol Res.

[14] Smith ME, Farah MJ (2011). Are prescription stimulants “smart pills”? The epidemiology and cognitive neuroscience of prescription stimulant use by normal healthy individuals. Psychol Bull.

[15] Weyandt LL, Oster DR, Marraccini ME, Gudmundsdottir BG, Munro BA, Rathkey ES (2016). Prescription stimulant medication misuse: where are we and where do we go from here?. Exp Clin Psychopharmacol.

[16] Schifano F, Catalani V, Sharif S, Napoletano F, Corkery JM, Arillotta D (2022). Benefits and harms of “smart drugs” (nootropics) in healthy individuals. Drugs.

[17] Pozzi M, Carnovale C, Peeters GGAM, Gentili M, Antoniazzi S, Radice S (2018). Adverse drug events related to mood and emotion in paediatric patients treated for ADHD: a meta-analysis. J Affect Disord.

[18] Bramness JG, Gundersen ØH, Guterstam J, Rognli EB, Konstenius M, Løberg E-M (2012). Amphetamine-induced psychosis—a separate diagnostic entity or primary psychosis triggered in the vulnerable?. BMC Psychiatry.

[19] Carlier J, Giorgetti R, Varì MR, Pirani F, Ricci G, Busardò FP (2019). Use of cognitive enhancers: methylphenidate and analogs. Eur Rev Med Pharmacol Sci.

[20] Faraone SV, Rostain AL, Montano CB, Mason O, Antshel KM, Newcorn JH (2020). Systematic review: nonmedical use of prescription stimulants: risk factors, outcomes, and risk reduction strategies. J Am Acad Child Adolesc Psychiatry.

[21] Tuttle JP, Scheurich NE, Ranseen J (2010). Prevalence of ADHD diagnosis and nonmedical prescription stimulant use in medical students. Acad Psychiatry.

[22] Webb JR, Valasek MA, North CS (2013). Prevalence of stimulant use in a sample of US medical students. Ann Clin Psychiatry.

[23] Kudlow PA, Naylor KT, Xie B, McIntyre RS (2013). Cognitive enhancement in Canadian medical students. J Psychoact Drugs.

[24] Lengvenytė A, Strumila R (2016). Do medical students use cognitive enhancers to study? Prevalence and correlates from Lithuanian medical students sample. Eur Psychiatry.

[25] Jain R, Chang CC, Koto M, Geldenhuys A, Nichol R, Joubert G (2017). Non-medical use of methylphenidate among medical students of the University of the Free State. S Afr J Psychiatr.

[26] Cohen YG, Segev RW, Shlafman N, Novack V, Ifergane G (2015). Methylphenidate use among medical students at Ben-Gurion University of the Negev. J Neurosci Rural Pract.

[27] Finger G, da Silva ER, Falavigna A (2013). Use of methylphenidate among medical students: a systematic review. Rev Assoc Med Bras.

[28] De Bruyn S, Wouters E, Ponnet K, Van Hal G (2019). Popping smart pills in medical school: are competition and stress associated with the misuse of prescription stimulants among students?. Subst Use Misuse.

[29] Kessler RC, Adler L, Ames M, Demler O, Faraone S, Hiripi E (2005). The World Health Organization Adult ADHD Self-Report Scale (ASRS): a short screening scale for use in the general population. Psychol Med.

[30] Ustun B, Adler LA, Rudin C, Faraone SV, Spencer TJ, Berglund P (2017). The World Health Organization adult attention-deficit/hyperactivity disorder self-report screening scale for DSM-5. JAMA Psychiat.

[31] Zohar AH, Konfortes H (2010). Diagnosing ADHD in Israeli adults: the psychometric properties of the adult ADHD Self Report Scale (ASRS) in Hebrew. Isr J Psychiatry Relat Sci.

[32] Fond G, Gavaret M, Vidal C, Brunel L, Riveline J-P, Micoulaud-Franchi J-A (2016). (Mis)use of prescribed stimulants in the medical student community: motives and behaviors: a population-based cross-sectional study. Medicine (Baltimore).

[33] Moore DR, Burgard DA, Larson RG, Ferm M (2014). Psychostimulant use among college students during periods of high and low stress: an interdisciplinary approach utilizing both self-report and unobtrusive chemical sample data. Addict Behav.

[34] Dussault CL, Weyandt LL (2013). An examination of prescription stimulant misuse and psychological variables among sorority and fraternity college populations. J Atten Disord.

[35] Verdi G, Weyandt LL, Zavras BM (2016). Non-medical prescription stimulant use in graduate students: relationship with academic self-efficacy and psychological variables. J Atten Disord.

[36] McManus B, Galbraith JW, Heaton K, Mrug S, Ponce BA, Porterfield JR (2020). Sleep and stress before and after duty across residency years under 2017 ACGME hours. Am J Surg.

[37] Kasi PM, Khawar T, Khan FH, Kiani JG, Khan UZ, Khan HM (2007). Studying the association between postgraduate trainees’ work hours, stress and the use of maladaptive coping strategies. J Ayub Med Coll Abbottabad.

[38] Dyrbye L, Shanafelt T (2016). A narrative review on burnout experienced by medical students and residents. Med Educ.

[39] Rosen IM, Gimotty PA, Shea JA, Bellini LM (2006). Evolution of sleep quantity, sleep deprivation, mood disturbances, empathy, and burnout among interns. Acad Med.

[40] Perez AR, Boscardin CK, Pardo M (2022). Residents’ challenges in transitioning to residency and recommended strategies for improvement. J Educ Perioper Med.

[41] Thomas NK (2004). Resident burnout. JAMA.

[42] Mirsham. 26-hour survey. https://www.mirsham.org.il/26-%D7%A9%D7%A2%D7%95%D7%AA/. Accessed 28 Jan 2022.

[43] Serenari M, Cucchetti A, Russo PM, Fallani G, Mattarozzi K, Pinna AD (2019). Burnout and psychological distress between surgical and non-surgical residents. Updates Surg.

[44] Christie JD, Rosen IM, Bellini LM, Inglesby TV, Lindsay J, Alper A (1998). Prescription drug use and self-prescription among resident physicians. JAMA.

[45] Montgomery AJ, Bradley C, Rochfort A, Panagopoulou E (2011). A review of self-medication in physicians and medical students. Occup Med (Lond).

[46] Kumar P, Basu D (2000). Substance abuse by medical students and doctors. J Indian Med Assoc.

[47] Zacay G, Baron-Epel O, Malatskey L, Heymann A (2021). Preferences and barriers to the utilization of primary health care by sick physicians: a nationwide survey. Fam Pract.

[48] Sudhinaraset M, Wigglesworth C, Takeuchi DT (2016). Social and cultural contexts of alcohol use: influences in a social-ecological framework. Alcohol Res.

[49] Feingold D, Goldberger N, Haklai Z, Lev-Ran S (2017). Fatal overdoses of opioids in Israel 2005–2014. Eur Addict Res.

[50] Lundgren L, Padyab M, Lucero NM, Blom-Nilsson M, Nyström S, Carver-Roberts T (2019). Immigration status and substance use disorder-related mortality in Sweden: a National Longitudinal Registry study. J Addict Med.

