# Supplementary material for: Methylphenidate use and misuse among medical residents in Israel: a cross-sectional study
Source: Hum Resour Health. 2023 Jan 31;21:5. doi: 10.1186/s12960-023-00792-x (PMC9890881; doi:10.1186/s12960-023-00792-x)
Supplement: Supplementary file 1 — Additional file 1. Stimulant medication use among Israeli medical residents: questionnaire. [file 12960_2023_792_MOESM1_ESM.docx]

**Stimulant Medication Use among Israeli Medical Residents: Questionnaire**

**Demographic characteristics**

1) Age:

2) Gender: **M/F**

3) Birth country: **Israel/Other**

) Native language**: Hebrew/Other**4

5) Number of children during residency examination period: **none/1/2+**

6) Residency type:

**internal medicine/pediatric/family medicine/OB-GYN/surgical (general, pediatric, plastic, ENT, orthopedics, urology)/other (neurology, radiology, etc.)**

**ASRS (Adult ADHD Self-Report Scale)**

Please answer the questions below, rating yourself on each of the criteria shown using the scale. As you answer each question, place an X in the box that best describes how you have felt and conducted yourself over the past 6 months.

1-never, 2-rarely, 3-sometimes, 4-often, 5-very often

How often do you have trouble wrapping up the final details of a project once you have completed the challenging parts?

**1/2/3/4/5**

How often do you have difficulty getting things in order when you are required to carry out a task that requires organization?

**1/2/3/4/5**

How often do you have problems remembering appointments or obligations?

**1/2/3/4/5**

When you have a task that requires a lot of thought, how often do you avoid it or delay getting started?

**1/2/3/4/5**

How often do you fidget or squirm with your hands or feet when you have to sit down for a long time?

**1/2/3/4/5**

How often do you feel overly active and compelled to do things, as if you are driven by a motor?

**1/2/3/4/5**

**Stimulant use**

1) Have you ever been formally diagnosed with ADD/ADHD? **Yes No**

2) If you answered yes to the previous question, when were you diagnosed? Mark the most accurate answer.

**primary school/high school/before or during academic studies/residency**

3) Have you ever used methylphenidate (Ritalin, concerta, etc.) or any other stimulant drug (amphetamine salts such as Vyvanse, Attent, Focalin, etc.) beyond experimentation? **Yes/No**

4) If you answered yes to the previous question, when did you first use it? Mark the most accurate answer.

**primary school/high school/medical school-pre clinical years/medical school-clinical years/residency**

5) How familiar are you with the safety and adverse effect profiles of stimulant drugs (1–5: 1-not at all, 5-very well)

**1/2/3/4/5**

**Self-approach towards the use of stimulant drugs:**

Please answer the questions below, rating yourself on each of the criteria shown using the scale.

1- completely disagree, 2- mainly disagree, 3- I have no opinion on the matter, 4- mainly agree, 5- completely agree

1) I object to the use of drugs for cognitive enhancement and academic performance improvement, for example, a law student without an ADD/ADHD diagnosis using Ritalin while studying for final exams.

**1/2/3/4/5**

2) I believe that the use of stimulant drugs (such as methylphenidate) without an ADD/ADHD diagnosis by only some of the students taking an exam detracts from the fairness and equity of the exam (similarly to an athlete’s use of a performance-enhancing drug before an important competition).

**1/2/3/4/5**

3) Stimulant drugs (such as methylphenidate) should be prescribed only in situations in which the patient is incapable of handling basic organizational and learning tasks without the medication and the negative impact on his quality of life is substantial.

**1/2/3/4/5**

4) The initial prescription of stimulant drugs should be given only by an ADD/ADHD specialist and only after a formal diagnosis.

**1/2/3/4/5**

5) Any change in stimulant drug prescriptions, including dosage or alternative medication, should be made only by a specialist on the subject.

**1/2/3/4/5**

**Use of stimulant drugs during residency**

1) Did you use a stimulant drug at any time during residency (beyond experimentation)? **Yes/ No**

# Only for subjects who used stimulant drugs during residency:

2) In what situations have you used stimulant drugs? Mark all the relevant answers.

**studying for residency examination/night shifts/daily use/other: ___________**

3) For those who used stimulant drugs when studying for the final residency exam: How frequently did you use the drug?

**Daily use for more than a month/daily use for less than a month/occasionally on an as-needed basic (more than 10 days total)/occasionally on an as-needed basic (less than 10 days total)**
